# Serum uric acid and cardiovascular mortality in chronic kidney disease: a meta-analysis

**DOI:** 10.1186/s12882-018-1143-7

**Published:** 2019-01-14

**Authors:** Qimei Luo, Xi Xia, Bin Li, Zhenchuan Lin, Xueqing Yu, Fengxian Huang

**Affiliations:** 1 Department of Nephrology, The First Affiliated Hospital of Sun Yat-sen University, Guangzhou, 510080 People’s Republic of China; 2 Key Laboratory of Nephrology, Ministry of Health and Guangdong Province, Guangzhou, 510080 People’s Republic of China

**Keywords:** Chronic kidney disease, Meta-analysis, Cardiovascular mortality, Serum uric acid

## Abstract

**Background:**

Conflicting results have been reported from studies evaluating serum uric acid (SUA) levels as an independent risk factor for cardiovascular mortality in patients with chronic kidney disease (CKD).

**Methods:**

We systematically searched MEDLINE, Web of Science, and bibliographies of retrieved articles to identify studies reporting on the association between SUA levels and cardiovascular mortality in patients with CKD. Random-effects models were used to calculate the pooled hazard ratios (HR) and corresponding 95% confidence intervals (CI).

**Results:**

We included 11 studies with an overall sample of 27,081 patients with CKD in this meta-analysis. By meta-analysis, restricted to 7 studies (*n* = 11,050), patients with the highest SUA were associated with an increased risk of cardiovascular mortality (HR 1.47, 95% CI 1.11–1.96) compared with patients with the lowest SUA. There was no indication of publication bias or significant heterogeneity (*I*^2^ = 40.4%; *P* = 0.109). Meta-analysis of 10 studies (*n* = 26,660) indicated that every 1 mg/dl increase in SUA levels increased a 12% risk in cardiovascular mortality (HR 1.12, 95% CI 1.02–1.24), with significant heterogeneity (*I*^2^ = 79.2%, *P* < 0.001).

**Conclusions:**

Higher SUA levels are associated with significantly increased risk of cardiovascular mortality in patients with CKD. More designed studies, especially randomized controlled trials, should be conducted to determine whether high SUA levels is a potentially modifiable risk factor for cardiovascular mortality in patients with CKD.

**Electronic supplementary material:**

The online version of this article (10.1186/s12882-018-1143-7) contains supplementary material, which is available to authorized users.

## Background

Chronic kidney disease (CKD) has become a major global health burden. The age-standardized global prevalence of CKD stages 1–5 among adults in 2010 was 10.4% in men and 11.8% in women [1]. CKD was ranked as the 19th highest global cause of life years lost, with an age-standardized death rate of 15.8 per 100,000 [2]. Patients with CKD have an increased risk of cardiovascular mortality compared with those with normal kidney function [3]. Cardiovascular disease (CVD) causes most of the deaths in individuals with CKD [3, 4]. Many risk factors have been identified for cardiovascular mortality in patients with CKD, such as smoking, diabetes, systolic hypertension, high levels of C-reactive protein, and left ventricular hypertrophy [5–7]. Several studies have suggested that hyperuricemia may be another potential risk factor for cardiovascular mortality in individuals with CKD [8–10]. Elevated serum uric acid (SUA) is related to the development and progression of hypertension [11], stroke [12], and cardiovascular disease [13]. In the general population, SUA level is an independent predictor for future cardiovascular mortality [14].

Many [8, 9, 15–18] but not all [19, 20] studies have detected a significant association between SUA levels and cardiovascular mortality in individuals with CKD. Furthermore, there is still some debate whether SUA levels can be an independent risk factor for cardiovascular mortality in patients with CKD. Several studies [8, 9, 16] indicated that higher SUA is a predictor of increased risk of death from cardiovascular disease in patients with CKD, while other studies [15, 17, 18] reported that higher SUA levels are associated with lower risk of cardiovascular mortality. We hypothesized that SUA levels could be a predictor for cardiovascular mortality in individual with CKD. In this study, we conduct a meta-analysis of all relevant studies to evaluate the association of SUA levels with cardiovascular mortality in patients with CKD.

## Methods

### Study design and search strategy

The meta-analysis was designed and conducted according to the consensus guidelines in “Meta-Analysis of Observational Studies in Epidemiology” [21]. A systematic literature search was performed using the PubMed and Web of Science databases from the time of their inception to May 8, 2017. No restrictions were imposed on the search. The following search terms and search strings were used: (“uric acid” OR hyperuricemia OR urate) AND (renal or kidney or dialysis) AND (mortality or death or survival or cardiovascular or stroke). The key words and abstract were searched. In addition, we performed a manual survey of cited references in the retrieved reviews and articles to capture all relevant studies. Two of the authors independently conducted the literature searches.

### Study selection

The following inclusion criteria were used to select studies for this meta-analysis: (1) studies had baseline information about SUA concentration; (2) studies exploring cardiovascular mortality as a study outcome; (3) studies recruiting adult patients with CKD and CKD defined according to the Kidney Disease Outcomes Quality Initiative (KDOQI) guideline [22]; and (4) studies that compared the highest SUA levels with the lowest SUA levels, or performed controlled comparison of 1 mg/dl increase in SUA levels, and provided statistical data on hazard ratio (HR) and 95% confidence interval (CI), or included sufficient data to enable the calculation of HR and 95% CI. In the case of multiple studies based on the same population or subpopulation, only the most informative report or the most recent publication was included. Two authors independently reviewed the retrieved articles and determined whether the article satisfied the inclusion criteria. Disagreement was resolved by discussion with the senior author. We requested additional data from corresponding authors for two articles (*n* = 2) to enable statistical calculation of HR and 95% CI. However, the corresponding authors in both cases did not respond to our requests. A total of 44 articles were selected by the inclusion criteria, and these were then subjected to the exclusion criteria listed in Additional file 1: Table S1. Finally, a total of 11 articles were selected for this meta-analysis.

### Data extraction and quality assessment

Two authors independently extracted data from full-text articles. The data included author, publication year, study design, population, number of participants, mean age of participants, percentage of men, diabetes, mean SUA levels, median duration of follow-up, number of deaths due to cardiovascular events, definition of cardiovascular mortality, and adjusted factors. The most fully adjusted HR and 95% CI were extracted from all included studies. The quality of observational cohort studies was evaluated according to the Newcastle-Ottawa quality assessment scale [23]. Study quality was categorized as poor (score 1–3), fair (score 4–6), or good (score 7–9) depending on the quality of study participant selection, comparability, and outcome. The quality of randomized controlled trials was evaluated using the Jadad scale; a high-quality trial should have a Jadad score ≥ 3 [24].

### Statistical analysis

HRs were adjusted for the maximum number of confounding variables. Every HR was transformed into the natural logarithm (log HR), and the standard error obtained from its corresponding 95% CI. Since multivariable Cox proportional hazards regression analyses were used to obtain the adjusted HRs of SUA levels for cardiovascular mortality, random-effects analyses were performed to calculate the pooled log HR values. Then, we obtained the overall HR and its corresponding 95% CI through exponentiation of the pooled log HR [25].

We analyzed SUA as a continuous variable to test the possibility of a nonlinear dose-response relationship between SUA levels and cardiovascular mortality [26]. For this analysis, we used the restricted cubic splines method with three knots at the 25th, 50th, and 75th percentiles of serum uric levels. In some cases, SUA was analyzed as a categorical variable to evaluate the trends between SUA levels and cardiovascular mortality using two different methods. Generalized least-squares regression analysis was used when data for the cases and person-years or participants and HRs were available for each category. Variance-weighted least-squares regression analysis was used when information for the cases or participants was not available for each category [27, 28].

The *I*^2^ index, *Q* statistic, and *P* value were used to describe the heterogeneity of effect size estimates across studies. We performed meta-regression analyses to evaluate the influence of prespecified covariates on between-study heterogeneity. Funnel plots were computed and Egger’s tests were performed to assess the potential publication bias [29]. Sensitivity analyses were performed to evaluate the influence of a single study on the overall risk estimate by removing one study at a time. All analyses were performed with STATA statistical software version 12.0 (Stata Corp, College Station, TX, USA). A *P* value < 0.05 was regarded as statistically significant.

## Results

### Study selection and study characteristics

**Fig. 1:**
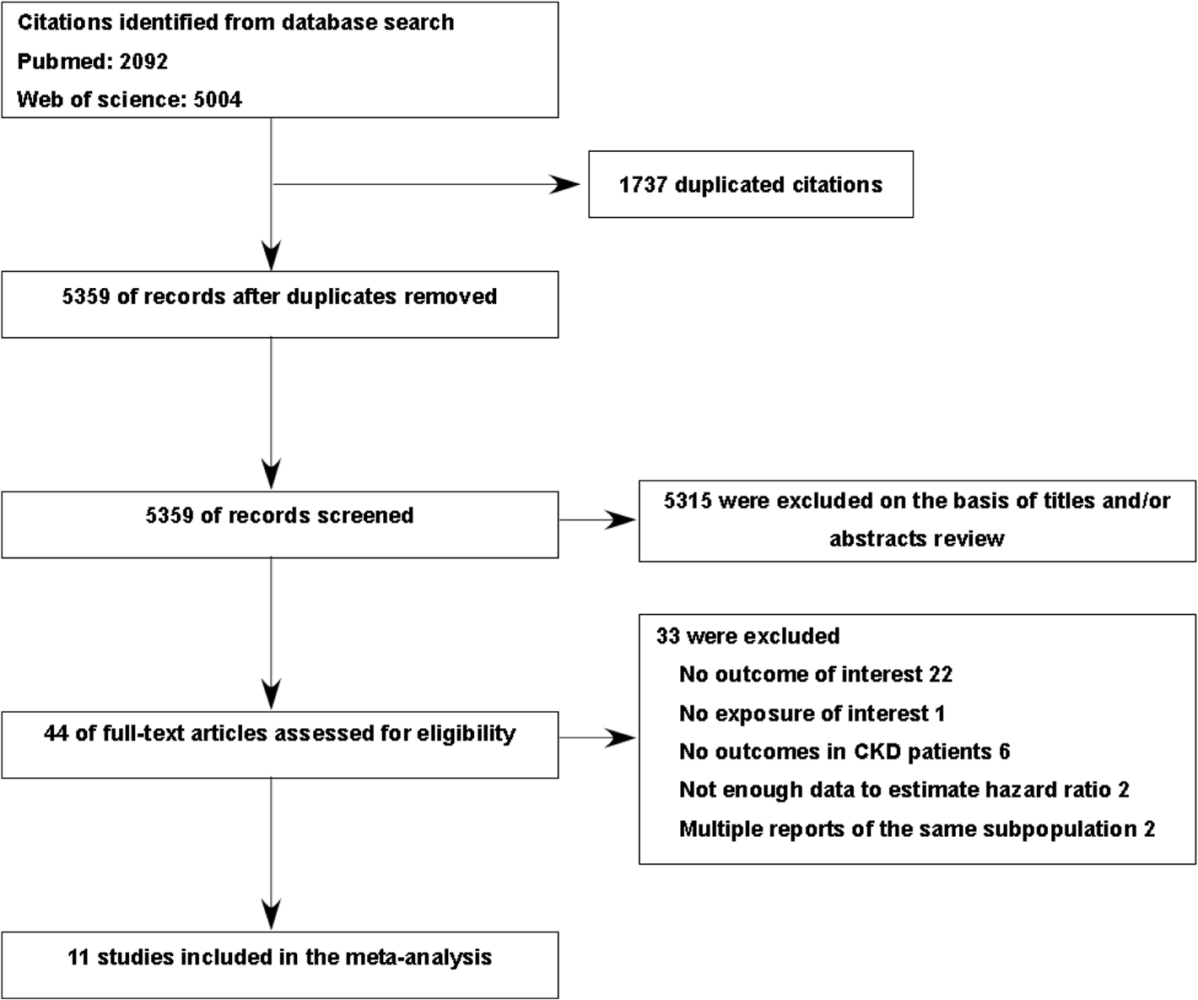
Flow chart of study selection of included studies. Abbreviation: CKD, chronic kidney disease

Our systematic literature search was inclusive from the database inception up to May 8, 2017. We retrieved 2.092 abstracts in PubMed and 5004 abstracts in Web of Science. A total of 5359 non-duplicate studies were identified. After the first selection procedure, we evaluated 44 full-text articles further. Finally, 11 articles representing 27,081 study participants fulfilled all eligibility criteria [8–10, 16–20, 30–32]. A detailed description of our study selection is presented in Fig. 1. The characteristics and quality of the individual studies are presented in Table 1. The articles included four prospective cohort studies, six retrospective cohort studies, and one randomized controlled trial. The number of participants in individual studies ranged from 261 [30] to 13,059 [16]. The percentage of study participants with diabetes varied from 5% [8] to 59% [30]. The median duration of follow-up ranged from 23 months to 10 years. All 11 studies provided adjusted risk estimates, nine studies adjusted for kidney function [8–10, 16, 18–20, 30, 31], and five studies adjusted for drugs to lower uric acid levels [8, 10, 17, 18, 31].

**Table 1:** Characteristics of 11 studies included in the meta-analysis

Author, year	Study design	Population	Patients (n)	Men (%)	Diabetes (%)	Age (years)	Uric acid (mg/dl)	Follow-up	Cardiovascular mortality events and definition (n)	Comparison	Adjust HR (95%CI)	Adjustments	Quality score
Madero, 2009 [8]	RCT	United States, MDRD, CKD3–4	838	39	5	52 ± 12	7.63 ± 1.66	Median 10 years	127 CV mortality was defined as death resulting from CVD (International classification of diseases, ninth revision [ICD-9] codes 390 to 459)	Per 1 mg/dl increase Tertile 3 vs. tertile 1 8.4–15.6 mg/dl vs. 1.7–6.9 mg/dl	1.16 (1.01–1.33) 1.47 (0.90–2.39)	Age, gender, blood pressure, protein diet randomization assignments, history of CVD, DM, BMI, systolic blood pressure, HDL-C, log-transformed C-reactive protein, GFR, albumin, diuretic, allopurinol.	3
Latif, 2011 [17]	PCS	DOPPS, HD	4637	58.2	24.5	Mean 58–64	Mean 6.97	Median 23 months	NA CV mortality was defined as death resulting from acute myocardial infarction, atherosclerotic heart disease, cardiomyopathy, cardiac arrhythmia, cardiac arrest, congestive heart failure, cerebrovascular accident including intracranial hemorrhage, ischemic brain damage and anoxic encephalopathy	Per 1 mg/dl increase ≦8.2 mg/dl vs. > 8.2 mg/dl	0.92 (0.86,0.99) 1.54 (1.15–2.07)	Age, black race, gender, BMI, years with ESRD, albumin-corrected calcium, albumin, ferritin, creatinine, phosphorus, allopurinol, 14 comorbid conditions, study phase and facility	5
Kanbay, 2012 [9]	RCS	Turkey, CKD 3–5	303	49.8	23.4	Mean 47–53	NA	Median 39 months	33 CV mortality was defined as death resulting from coronary heart disease, sudden death, stroke and complicated peripheral vascular disease	Per 1 mg/dl increase	2.819 (1.783–4.458)	Age, gender, eGFR, DM, smoking, hypertension, LDL, systolic blood pressure, hsCRP, HOMA-IR index, FMD, NMD	6
Kuo, 2013 [16]	RCS	Taiwan, CKD	13,059	NA	NA	NA	NA	Median 4.6 years	NA CV mortality was defined as death resulting from cardiovascular disease (ICD-9 codes: 390.x-459.x)	9.0–10.9 mg/dl vs. 5.0–6.9 mg/dl ≧11.0 mg/dl vs. 5.0–6.9 mg/dl	1.42 (1.11–1.81) 1.65 (1.22–2.24)	Age, gender, eGFR, fasting glucose, total cholesterol and history of hypertension, DM, CHD, stroke, heart failure.	6
Yin, 2013 [20]	RCS	China, CKD undergoing DES	1132	71.7	33.6	67.7 ± 7.8	7.8 ± 1.9	Median 38.5 months	50 CV mortality was defined as death resulting from coronary artery disease, cardiac arrhythmia, congestive heart failure, sudden death	Quartile 4 vs. quartile 1 > 8.98 mg/dl vs. < 6.46 mg/dl	0.84 (0.37–1.89)	Age, gender, DM, eGFR, left ventricular ejection, proteinuria, AMI, incomplete revascularization	7
Dong, 2014 [19]	RCS	China, SSOP, PD	2193	49	37.7	58.1 ± 15.5	6.41 ± 1.87	Median 26.5 months	231 CV mortality was defined as death resulting from myocardial infarction, congestive heart failure, cerebral bleeding, cerebral infarction, arrhythmia, peripheral arterial disease and sudden death	Per 1 mg/dl increase Gender-specific Tertile 3 vs. tertile 1 Men: 7.39–16.7 mg/dl women: 6.66–8.08 mg/dl vs. Men: 2.09–5.79 mg/dl women: 1.74–5.37 mg/dl	1.04 (0.89,1.20) 1.35 (0.74,2.46)	Age, residual renal function, albumin, hemoglobin, phosphate, C-reactive protein, history of CVD, DM, BMI, mean arterial pressure, LDL-C, center size, gender-adjusted only SUA as continuous variable	7
Miyaoka, 2014 [18]	RCS	Japan, CKD2–4	551	59.3	10	58.5	6.57 ± 1.35	6 years	19 CV mortality was defined as death resulting from myocardial infarction, congestive heart failure.	Tertile 3 vs. tertile 1 7.2–12.4 mg/dl vs. 3.0–5.8 mg/dl Hyperuricemia vs. normouricemia Hyperuricemia (> 7.0 mg/dl or with allopurinol)	1.042 (0.139–7.831) 0.274 (0.103–0.731)	Gender, smoking status, history of CVD, systolic blood pressure, HDL-C, triglyceride, hemoglobin, C-reactive protein (log), eGFR, proteinuria (log), etiology of kidney disease, diuretics, allopurinol Gender, smoking status, CVD, HDL-C, triglyceride, hemoglobin, C-reactive protein (log), eGFR, proteinuria (log), etiology of kidney disease, diuretics, allopurinol	8
Beberashvili, 2015 [30]	PCS	Isreael, MHD	261	61.3	59	68.6 ± 13.6	5.76 ± 1.16	2 years	31 CV mortality was defined as death resulting from coronary heart disease, sudden death, stroke, or complicated peripheral vascular disease.	Per 1 mg/dl increase	0.53 (0.33–0.86)	Age, gender, vintage, Kt/v, DM, comorbidity index, smoking, systolic blood pressure, waist hip rate, phosphorus, creatinine, residual renal function, malnutrition inflammation score, interleukin-6	7
Hsieh, 2015 [31]	RCS	Taiwan CKD 3–5	2408	56.9	38.3	65.7 ± 12.6	7.73 ± 1.78	Median 3.03 years	143 CV mortality was defined as death resulting from coronary artery disease, cerebrovascular or peripheral vascular disease	Per 1 mg/dl increase	1.16 (0.92–1.32)	Age, gender, BMI, DM, hypertension, cardiovascular disease, gout, glycated hemoglobin, cholesterol, triglyceride, BUN, eGFR, GPT, albumin, Ca × P, white blood cell count, hemoglobin, proteinuria, diuretics, hypouricemic agents, erythropoiesis stimulating agents, ACE inhibitor and angiotensin II receptor blocker	7
Xia, 2016 [10]	PCS	China, PD	1278	58.8	25.7	47.6 ± 15.0	7.2 ± 1.4	Median 30.7 months	126 CV mortality was defined as death resulting from acute myocardial infarction, cardiac arrhythmia, cardiac arrest, congestive heart failure, cerebrovascular accident, and peripheral vascular disease.	Per 1 mg/dl increase Gender-specific Tertile 3 vs. tertile 1 DM Men: 7.1–13.8 mg/dl, women: 6.9–12.6 mg/dl vs. Men: 1.5–5.6 mg/dl, women: 1.2–5.4 mg/dl. NDM Men: 7.1–13.8 mg/dl, women: 6.9–12.6 mg/dl vs. Men: 1.5–5.6 mg/dl, women: 1.2–5.4 mgj/dl	1.42 (1.13–1.79) DM Men 1.12 (0.78–1.61) DM Women 1.41 (1.09–1.82) NDM Men 1.24 (0.85–1.82) NDM Women 2.26 (1.14–4.48) DM 3.07 (1.54–6.08) NDM	Age, BMI, hypertension, CVD, hemoglobin, albumin, phosphorus, serum creatinine, HDL-C, residual renal function, log-transformed high-sensitive C-reactive protein, glycated hemoglobin, use of allopurinol, and use of ACE inhibitor or angiotensin receptor blocker. Gender-adjusted only SUA as continuous variable. Glycated hemoglobin-adjusted on in DM.	8
Li, 2016 [32]	PCS	China, CKD 3–5	421	NA	NA	NA	NA	Median 3.9 years	NA	Tertile 4 vs. tertile 1 7.29–18.88 mg/dl vs ≤ 5.08 mg/dl	0.72 (0.28,1.81)	Age, gender, leisure-time physical activity, smoking, alcohol drinking, occupation, BMI, SBP, DBP, LDL-C, duration of CAD, type of CAD, history of diabetes, history of heart failure, coronary artery stenosis degree on coronary angiography, use of antidiabetic, cholesterol-lowering or antiplatelet drugs, use of diuretics, β-blockers and antihypertensive drugs	6

Conversion factors for units: serum uric acid in mg/dl to umol/l, ×59.48

Abbreviations: *AMI* acute myocardial infarction, *BMI* body mass index, *CAD* coronary artery disease, *CV* cardiovascular, *CVD* cardiovascular disease, *CHD* coronary heart disease, *CKD* chronic kidney disease, *DBP* diastolic blood pressure, *DM* diabetes mellitus, *DOPPS* The Dialysis Outcome and Practice Patterns Study, *DES* drug-eluting stent, *eGFR* estimated glomerular filtration rate, *ESRD* end-stage renal disease, *FMD* flow-mediated dilatation, *HD* hemodialysis, *HDL-C* high density lipoprotein cholesterol, *HOMA-IR* homeostasis model assessment-insulin resistance, *hsCRP* high sensitivity C reactive protein, *LDL-C* low density lipoprotein cholesterol, *MHD* maintenance hemodialysis, *MDRD* Modification of Diet In Renal Disease, *NDM* nondiabetes, *NMD* nitroglycerine-mediated dilatation, *NA* not available, *PD* peritoneal dialysis, *SBP* systolic blood pressure, *SSOP* Socioeconomic Status on the Outcome of Peritoneal Dialysis, *SUA* serum uric acid, *RCT* randomized controlled trial, *PCS* prospective cohort study, *RCS* retrospective cohort study

The definition of cardiovascular mortality events varied among the included studies: two studies used the International Classification of Diseases Codes (ICD) [8, 16]; five studies reported the mortality from cardiovascular disease, cerebrovascular disease, and peripheral vascular disease [9, 10, 19, 30, 31]; one study reported the mortality from cardiovascular disease and cerebrovascular disease [17]; two studies only reported the mortality from cardiovascular disease [18, 20]; and one study did not provide information regarding the definition of cardiovascular mortality [32]. None of the included studies reported the risk for cardiovascular mortality associated with the lowest and median SUA levels. All cohort studies had fair to good quality according to the Newcastle-Ottawa Scale. The randomized control trial had high quality with a Jadad score of 3.

### Association of SUA with cardiovascular mortality

**Fig. 2:**
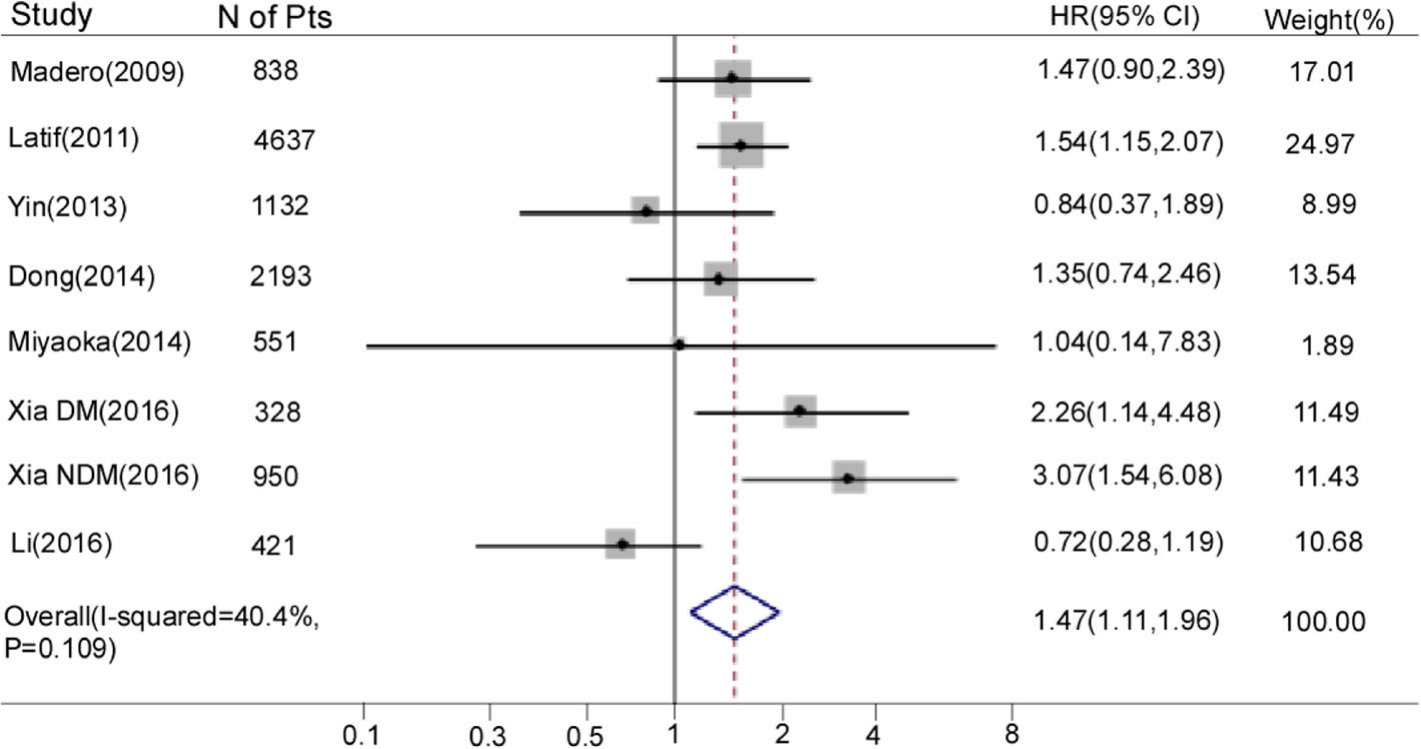
Forest plot and summary hazard ratio for the association of serum uric acid as a category variable and cardiovascular mortality in patients with chronic kidney disease. Abbreviations: CI, confidence interval; DM, diabetes mellitus; HR, hazard ratio; NDM, non-diabetes mellitus

1sSeven studies [8, 10, 17–20, 32] reported the association of SUA as a categorical variable with cardiovascular mortality. A total of 11,050 participants were included in this meta-analysis. Patients in the group with the highest SUA levels were associated with an increased risk of cardiovascular mortality compared with patients with the lowest levels (HR 1.47, 95% CI 1.11–1.96; Fig. 2). There was moderate heterogeneity among these included studies (*I*^2^ = 40.4%, *P* = 0.109). One study also reported the estimated association between SUA as the categorical variable and cardiovascular mortality [16]. Those authors reported that the adjusted HR for cardiovascular mortality was 1.42 (95% CI 1.11–1.81) and 1.65 (95% CI 1.22–2.24) for patients with the second-highest SUA levels (9.0–10.9 mg/dl) and those with the highest levels (≥11.0 mg/dl) compared with those with medium levels (5.0–6.9 mg/dl) [16].

**Fig. 3:**
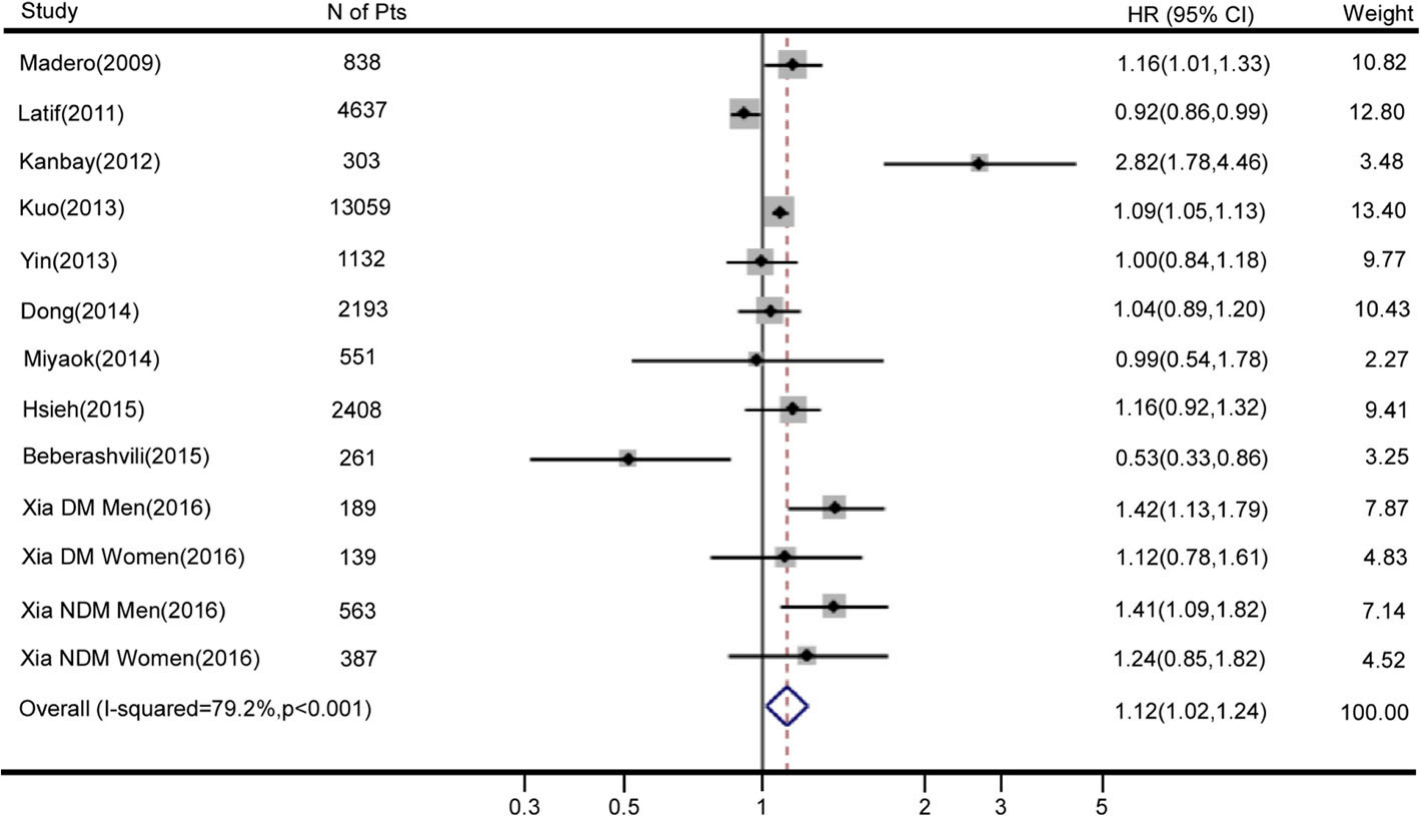
Forest plot and summary hazard ratio for the association of serum uric acid as a continuous variable (per 1 mg/dl increment) and cardiovascular mortality in patients with chronic kidney disease. Abbreviations: CI, confidence interval; DM, diabetes mellitus; HR, hazard ratio; NDM, non-diabetes mellitus

Seven of the included studies [8–10, 17, 19, 30, 31] reported the association of increasing SUA levels (per 1 mg/dl) with cardiovascular mortality. Three studies only reported the association of SUA as a categorical variable with cardiovascular mortality [16, 18, 20]; these results were evaluated using variance-weighted least-squares regression analyses to obtain the dose-response relationship between SUA levels and cardiovascular mortality. A total of 26,660 participants were included in this meta-analysis. Higher SUA levels (per 1 mg/dl increment) were associated with significantly higher hazard ratios for cardiovascular mortality (HR 1.12, 95% CI 1.02–1.24; Fig. 3). A significant heterogeneity among these studies was observed (*I*^2^ = 79.2%; *P* < 0.001). The nonlinear relationship test result suggested that SUA levels had no nonlinear association with cardiovascular mortality (*p* for nonlinearity = 0.109).

### Subgroup analyses

**Fig. 4:**
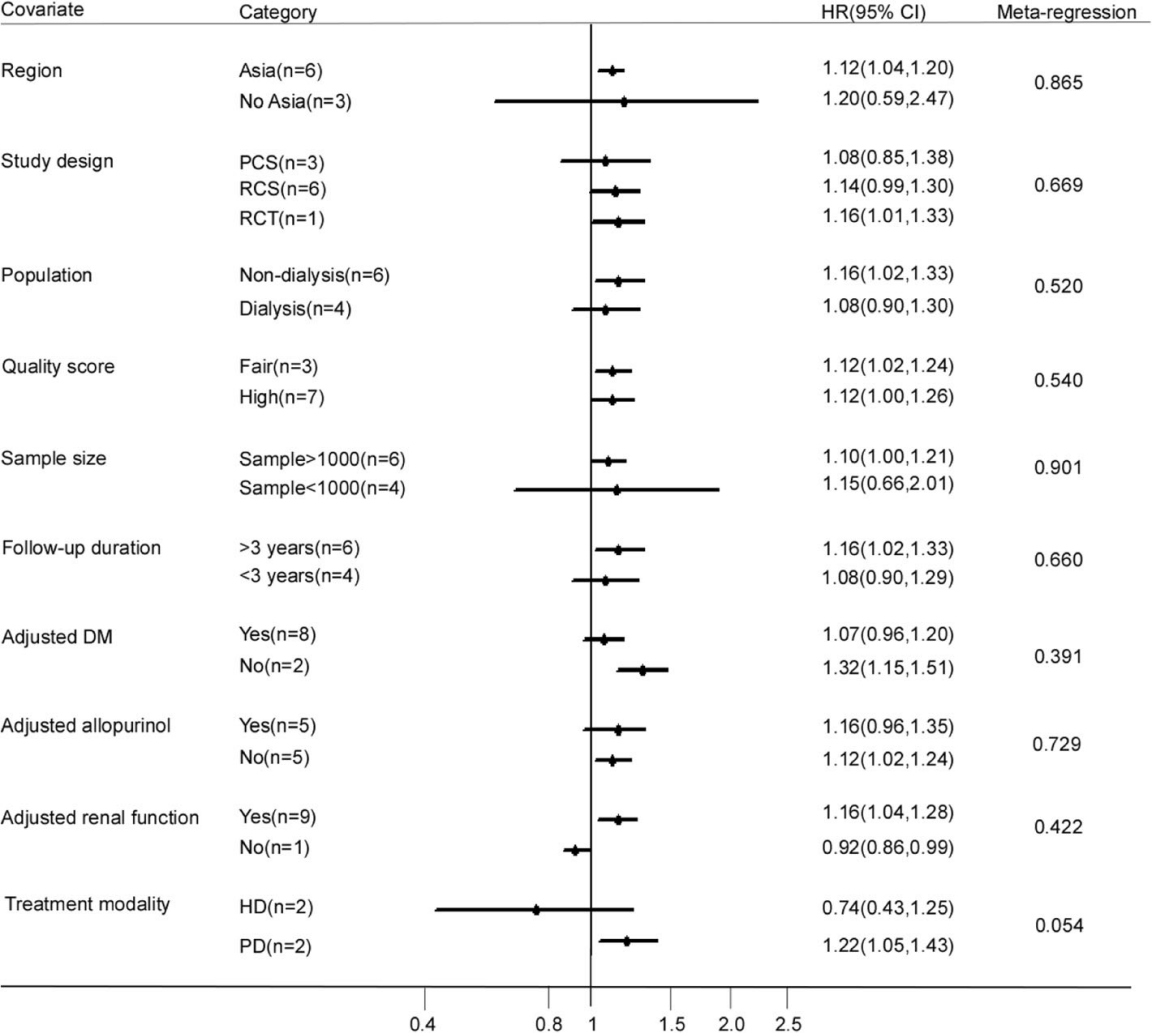
Forest plot and summary hazard ratio of subgroup analysis for the association of serum uric acid as a continuous variable (per 1 mg/dl increment) and cardiovascular mortality in patients with chronic kidney disease. Abbreviations: CI, confidence interval; DM, diabetes mellitus; HD, hemodialysis; HR, hazard ratio; NDM, non-diabetes mellitus; PD, peritoneal dialysis; PCS, prospective cohort study; RCT, randomized controlled trail; RCS, retrospective cohort study

Subgroup analyses were performed on the association of SUA (per 1 mg/dl increase) with cardiovascular mortality (Fig. 4). A significant and positive association between SUA levels and increased risk of cardiovascular mortality was observed in the Asian group, high quality score group, sample size > 1000 participants group, median follow-up duration > 3 years group, and adjusted for allopurinol treatment group. Six studies analyzed CKD populations that were not undergoing dialysis; each 1 mg/dl increase in SUA was independently associated with a 16% increase in cardiovascular mortality risk (HR 1.16, 95% CI 1.02–1.33). By contrast, for CKD populations that were undergoing dialysis, each 1 mg/dl increase in SUA was not associated with increased cardiovascular mortality risk (HR 1.08, 95% CI 0.90–1.29). Nine studies analyzed the association adjusted for kidney function, each 1 mg/dl increase in SUA was independently associated with a 16% increase in cardiovascular mortality risk (HR 1.16, 95% CI 1.04–1.28). Two cohort studies focused on patients receiving peritoneal dialysis (PD), and reported a significant association between higher SUA levels and increased cardiovascular mortality (HR 1.22, 95% CI 1.05–1.43). By contrast, two cohort studies focused on patients receiving hemodialysis (HD) did not observe any association between higher SUA levels and cardiovascular mortality (HR 0.74, 95% CI 0.43–1.25).

### Sensitivity analyses and publication bias

Sensitivity analyses and Egger’s tests for publication bias were performed using SUA as both categorical and continuous variables. The sensitivity analysis of SUA as a categorical variable indicated that there was moderate influence on the pooled HR and 95% CI when the study of Latif et al. [17] was excluded from the analysis. The sensitivity analysis of SUA as a continuous variable suggested that the results did not change significantly when one study was omitted at a time (Additional file 2: Figure S1). There was no statistical evidence of publication bias among studies of the association of SUA as a categorical variable and cardiovascular mortality (*p* = 0.680) or among studies of the association of SUA (per 1 mg/dl increase) with cardiovascular mortality (*p* = 0.430). The funnel plots representing these analyses are presented in Additional file 3: Figure S2.

## Discussion

Our meta-analysis of 11 cohort studies indicates that elevated SUA levels in patients with CKD are associated with significantly increased risk of cardiovascular mortality. This is the first meta-analysis that analyzed the association of SUA with cardiovascular mortality in patients with CKD. Our previous meta-analysis showed that higher SUA levels were associated with higher mortality risk in patients with CKD [33]. These results are consistent with the trends reported in three previous systematic reviews [12, 14, 34]. A meta-analysis of nine prospective observational studies with 165,922 participants reported that elevated SUA was associated with a 37% greater risk for cardiovascular mortality in the general population [14]. Two other meta-analyses focused on the association between hyperuricemia and mortality from stroke, and reported that hyperuricemia might increase the risk of stroke mortality [12, 34]. Previous meta-analysis reported that hyperuricemia increased the risk of cardiovascular events such as heart failure [35] and coronary heart disease [36]. Only one study reported this association separately in men and women [10], so we did not perform subgroup analysis for gender in our meta-analysis.

Two studies captured during our literature search were not included in this meta-analysis because they did not contain sufficient data to estimate the HR. A study with a cohort of 2645 patients with systolic heart failure analyzed the association of hyperuricemia with all-cause mortality, cardiovascular mortality, and heart failure hospitalization among all heart failure patients and those with and without CKD [37], and suggested that hyperuricemia was not significantly associated with cardiovascular mortality in heart failure patients with CKD. However, the study did not present detailed data for the HR of hyperuricemia with cardiovascular mortality. A single-center prospective study assessed whether SUA and superoxide dismutase could be predictive factors for cardiovascular mortality in patients undergoing hemodialysis [15], and found that lower SUA levels could predict cardiovascular mortality in hemodialysis patients (HR 0.937, 95% CI 0.883–0.994). However, this study analyzed the association of per 1 μmol/l increase in SUA levels with cardiovascular mortality, and did not provide sufficient data to calculate HR.

Several potential mechanisms support the pathogenic association between higher SUA levels and higher risk of cardiovascular mortality. Hyperuricemia could disturb mitochondrial function and reduce ATP content, which would induce oxidative stress [38]. Experimental studies have shown that uric acid could enhance NALP3 expression, caspase-1 activation, and IL-1β and ICAM-1 production, suggesting that uric acid could stimulate inflammatory responses [39, 40]. Uric acid itself also may increase inflammation in patients with CKD [41]. Higher uric acid levels are related with diminished endothelial function [42–44], and endothelial dysfunction may contribute to hypertension and cardiovascular morbidity [45]. Chronically elevated uric acid levels could cause structural changes in vessel walls [46].

The core aspects of hyperuricemia management were patient education, lifestyle advice, and pharmacological therapies such as allopurinol [47]. A prospective, randomized trial found that allopurinol treatment reduced risk of cardiovascular events in patients with CKD [48]. The results from an observational study suggested that allopurinol lowered cardiovascular-related mortality in Japanese patients receiving hemodialysis who had no history of cardiovascular disease [49]. A prospective study reported that treatment of hyperuricemia with allopurinol could improve endothelial function in patients with CKD [50].

Heterogeneity was significantly high among the 10 studies that analyzed the association of increasing SUA (per 1 mg/dl) with cardiovascular mortality. However, the results of meta-regression analyses indicated that region, study design, population, study quality, sample size, follow-up time, treatment modality, and adjustment for allopurinol treatment could not account for the heterogeneity. The high heterogeneity might be related to age and/or other participant characteristics across the analyzed studies. The definitions of cardiovascular mortality were not exactly the same among these 10 studies, which may partly explain the heterogeneity. The included studies all have different definitions of cardiovascular mortality, different entry stages of CKD, different definitions of high versus low uric acid, it seems pointless quoting HR for increased cardiovascular mortality given that the single number cannot be applied to any particular situation, but the results of our meta-analysis showed trend of higher uric acid and cardiovascular mortality in patients with CKD.

Subgroup analysis of the predictive role of SUA on cardiovascular mortality differed with respect to the treatment modality, which may be due to differences in the clearance of uric acid between hemodialysis and PD. Malnutrition in dialysis patients may lead to low uric acid levels. Higher uric acid in hemodialysis patients is an indicator of better nutritional status [17, 51], which may partly explain the association of lower uric acid levels with higher cardiovascular mortality. Few of the included studies explored the association between SUA and cardiovascular mortality with respect to different CKD stages. Therefore, there was not enough data to perform the subgroup analysis for CKD stage. Instead we performed the subgroup analysis for the population of non-dialysis or dialysis. A near-significantly larger effect (*p* = 0.054) of treatment modality was observed in subgroup analysis of pooled risk estimates. The heterogeneity among studies that analyzed the association of SUA as a categorical variable with cardiovascular mortality was quite low. Although there was no evidence of publication bias, it cannot be completely ruled out due to the unpublished null findings.

Several limitations of our study should be acknowledged. First, most of the included studies were observational studies that could not prove a causal relationship, and some potential confounders including declining kidney function, nutritional status, and urate-lowering therapy during follow-up were difficult to fully adjust. Second, high heterogeneity was observed among the included studies that analyzed the association of SUA as a continuous variable with cardiovascular mortality. Individual studies did not adjust for the same potential risk factors. Each study had different cut-off values for the SUA groups, which might lead to misclassification of individuals in each group. Third, although the nonlinear relationship test suggested an approximately linear relationship between SUA and cardiovascular mortality in patients with CKD, only three of the included studies provided sufficient data to perform the nonlinear relationship test. Therefore, more data is needed to support the exact relationship between SUA and cardiovascular mortality. Fourth, the sensitivity analysis of SUA as a categorical variable indicated that the results were not significant when the Latif [17] study was omitted. Fifth, only few of the included studies reported data for cardiovascular mortality with respect to gender; therefore, we could not evaluate whether SUA level affected the cardiovascular mortality risk of men and women.

Our study has several strengths. This study is the first systematic review and meta-analysis of the association of baseline SUA levels with cardiovascular mortality in patients with CKD. The included studies had relatively high quality and adjusted for other risk factors. Furthermore, we considered uric acid not only as a categorical variable but also as a continuous variable.

## Conclusions

In conclusion, the results of our meta-analysis suggest that elevated levels of SUA increase the risk of cardiovascular mortality in patients with CKD. Future studies, preferably randomized controlled studies, should explore whether hyperuricemia is a potentially modifiable risk factor for cardiovascular mortality in patients with CKD.

## Additional files

Additional file 1: **Table S1**. The reasons of excluded articles at the stage of eligibility. The list showed the reasons of excluded articles. (DOCX 86 kb)

Additional file 2: **Figure S1**. Sensitivity analysis. Sensitivity analyses were performed to evaluate the influence of a signal study on the overall risk estimate by removing one study at a time. (DOCX 13200 kb)

Additional file 3: **Figure S2**. Publication bias. Funnel plots and Egger’s test were performed to assess the potential publication bias. (DOCX 5726 kb)

## Abbreviations

CKD: Chronic kidney disease
CVD: Cardiovascular disease
HD: Hemodialysis
PD: Peritoneal dialysis
SUA: Serum uric acid

## References

[1] Mills KT, Xu Y, Zhang W (2015). A systematic analysis of worldwide population-based data on the global burden of chronic kidney disease in 2010. Kidney Int.

[2] Naghavi M, Wang H, Lozano R, et al. Global, regional, and national age-sex specific all-cause and cause-specific mortality for 240 causes of death, 1990–2013: a systematic analysis for the Global Burden of Disease Study 2013. Lancet (London, England). 2015;385(9963):117–71. 10.1016/S0140-6736(14)61682-2 PMC4340604 25530442

[3] Matsushita K, van der Velde M, Astor BC (2010). Association of estimated glomerular filtration rate and albuminuria with all-cause and cardiovascular mortality in general population cohorts: a collaborative meta-analysis. Lancet (London, England).

[4] Go AS, Chertow GM, Fan D, McCulloch CE, Hsu CY (2004). Chronic kidney disease and the risks of death, cardiovascular events, and hospitalization. N Engl J Med.

[5] Shlipak MG, Fried LF, Cushman M (2005). Cardiovascular mortality risk in chronic kidney disease: comparison of traditional and novel risk factors. JAMA.

[6] Orth SR, Hallan SI (2008). Smoking: a risk factor for progression of chronic kidney disease and for cardiovascular morbidity and mortality in renal patients--absence of evidence or evidence of absence?. Clin J Am Soc Nephrol.

[7] Nakamura K, Nakagawa H, Murakami Y (2015). Smoking increases the risk of all-cause and cardiovascular mortality in patients with chronic kidney disease. Kidney Int.

[8] Madero M, Sarnak MJ, Wang X (2009). Uric acid and long-term outcomes in CKD. Am J Kidney Dis.

[9] Kanbay M, Yilmaz MI, Sonmez A (2012). Serum uric acid independently predicts cardiovascular events in advanced nephropathy. Am J Nephrol.

[10] Xia X, Zhao C, Peng FF, et al. Serum uric acid predicts cardiovascular mortality in male peritoneal dialysis patients with diabetes. Nutr Metab Cardiovasc Dis. 2016;26(1):20–6. 10.1016/j.numecd.2015.10.011 26712272

[11] Grayson PC, Kim SY, LaValley M, Choi HK (2011). Hyperuricemia and incident hypertension: a systematic review and meta-analysis. Arthritis Care Res.

[12] Li M, Hou W, Zhang X, Hu L, Tang Z (2014). Hyperuricemia and risk of stroke: a systematic review and meta-analysis of prospective studies. Atherosclerosis.

[13] Kivity S, Kopel E, Maor E (2013). Association of serum uric acid and cardiovascular disease in healthy adults. Am J Cardiol.

[14] Zhao G, Huang L, Song M, Song Y (2013). Baseline serum uric acid level as a predictor of cardiovascular disease related mortality and all-cause mortality: a meta-analysis of prospective studies. Atherosclerosis.

[15] Antunovic T, Stefanovic A, Ratkovic M (2013). High uric acid and low superoxide dismutase as possible predictors of all-cause and cardiovascular mortality in hemodialysis patients. Int Urol Nephrol.

[16] Kuo CF, See LC, Yu KH, Chou IJ, Chiou MJ, Luo SF (2013). Significance of serum uric acid levels on the risk of all-cause and cardiovascular mortality. Rheumatology (Oxford, England).

[17] Latif W, Karaboyas A, Tong L (2011). Uric acid levels and all-cause and cardiovascular mortality in the hemodialysis population. Clin J Am Soc Nephrol.

[18] Miyaoka T, Mochizuki T, Takei T, Tsuchiya K, Nitta K (2014). Serum uric acid levels and long-term outcomes in chronic kidney disease. Heart Vessel.

[19] Dong J, Han Q-F, Zhu T-Y (2014). The associations of uric acid, cardiovascular and all-cause mortality in peritoneal Dialysis patients. PLoS One.

[20] Yin Z, Fang Z, Yang M, Du X, Nie B, Gao K (2013). Predictive value of serum uric acid levels on mortality in acute coronary syndrome patients with chronic kidney disease after drug-eluting stent implantation. Cardiology.

[21] Stroup DF, Berlin JA, Morton SC (2000). Meta-analysis of observational studies in epidemiology: a proposal for reporting. Meta-analysis of observational studies in epidemiology (MOOSE) group. JAMA.

[22] K/DOQI clinical practice guidelines for chronic kidney disease: evaluation, classification, and stratification. Am J Kidney Dis. 2002;39(2 Suppl 1):S1–266. 11904577

[23] Wells GA, Shea B, O’Connell D et al. The Newcastle-Ottawa (NOS) for assessing the quality of norandomised studies in meta-analysis http://www.ohri.ca/programs/clinical_epidemiology/oxford.asp.

[24] Jadad AR, Moore RA, Carroll D (1996). Assessing the quality of reports of randomized clinical trials: is blinding necessary?. Control Clin Trials.

[25] DerSimonian R, Laird N (1986). Meta-analysis in clinical trials. Control Clin Trials.

[26] Orsini N, Li R, Wolk A, Khudyakov P, Spiegelman D (2012). Meta-analysis for linear and nonlinear dose-response relations: examples, an evaluation of approximations, and software. Am J Epidemiol.

[27] Greenland S, Longnecker MP (1992). Methods for trend estimation from summarized dose-response data, with applications to meta-analysis. Am J Epidemiol.

[28] Orsini N, Bellocco R, Greenland S (2006). Generalized least squares for trend estimation of summarized dose-response data. Stata J.

[29] Egger M, Davey Smith G, Schneider M, Minder C (1997). Bias in meta-analysis detected by a simple, graphical test. BMJ.

[30] Beberashvili I, Sinuani I, Azar A et al: Serum uric acid as a clinically useful nutritional marker and predictor of outcome in maintenance hemodialysis patients. Nutrition (Burbank, Los Angeles County, Calif) 2015, 31(1):138–147. 10.1016/j.nut.2014.06.012 25466658

[31] Hsieh YP, Chang CC, Yang Y, Wen YK, Chiu PF, Lin CC. The role of uric acid in chronic kidney disease patients. Nephrology(Carlton). 2017;22(6):441–8. 10.1111/nep.12679 26610276

[32] Li Q, Zhang Y, Ding D (2016). Association between serum uric acid and mortality among Chinese patients with coronary artery disease. Cardiology.

[33] Xia X, Luo Q, Li B, Lin Z, Yu X, Huang F (2016). Serum uric acid and mortality in chronic kidney disease: a systematic review and meta-analysis. Metab Clin Exp.

[34] Kim SY, Guevara JP, Kim KM, Choi HK, Heitjan DF, Albert DA (2009). Hyperuricemia and risk of stroke: a systematic review and meta-analysis. Arthritis Rheum.

[35] Huang H, Huang B, Li Y (2014). Uric acid and risk of heart failure: a systematic review and meta-analysis. Eur J Heart Fail.

[36] Kim SY, Guevara JP, Kim KM, Choi HK, Heitjan DF, Albert DA (2010). Hyperuricemia and coronary heart disease: a systematic review and meta-analysis. Arthritis Care Res.

[37] Filippatos GS, Ahmed MI, Gladden JD (2011). Hyperuricaemia, chronic kidney disease, and outcomes in heart failure: potential mechanistic insights from epidemiological data. Eur Heart J.

[38] Cristobal-Garcia M, Garcia-Arroyo FE, Tapia E. Renal oxidative stress induced by long-term hyperuricemia alters mitochondrial function and maintains systemic hypertension. Oxid Med Cell Longev. 2015;2015:535686. 10.1155/2015/535686 PMC4396880 25918583

[39] Kosugi T, Nakayama T, Heinig M (2009). Effect of lowering uric acid on renal disease in the type 2 diabetic db/db mice. Am J Physiol Renal Physiol.

[40] Zazueta C, Johnson RJ, Lozada LG (2015). Soluble uric acid increases NALP3 inflammasome and interleukin-1beta expression in human primary renal proximal tubule epithelial cells through the toll-like receptor 4-mediated pathway. Oxidative Med Cell Longev.

[41] Prasad Sah OS, Qing YX (2015). Associations between hyperuricemia and chronic kidney disease: a review. Nephrourol Mon.

[42] Choi YJ, Yoon Y, Lee KY (2014). Uric acid induces endothelial dysfunction by vascular insulin resistance associated with the impairment of nitric oxide synthesis. FASEB J.

[43] Hong Q, Qi K, Feng Z (2012). Hyperuricemia induces endothelial dysfunction via mitochondrial Na+/Ca2+ exchanger-mediated mitochondrial calcium overload. Cell Calcium.

[44] Li P, Zhang L, Zhang M, Zhou C, Lin N (2016). Uric acid enhances PKC-dependent eNOS phosphorylation and mediates cellular ER stress: a mechanism for uric acid-induced endothelial dysfunction. Int J Mol Med.

[45] Tang Z, Cheng LT, Li HY, Wang T (2009). Serum uric acid and endothelial dysfunction in continuous ambulatory peritoneal dialysis patients. Am J Nephrol.

[46] Kanbay M, Segal M, Afsar B, Kang DH, Rodriguez-Iturbe B, Johnson RJ (2013). The role of uric acid in the pathogenesis of human cardiovascular disease. Heart.

[47] Grassi D, Pontremoli R, Bocale R, Ferri C, Desideri G (2014). Therapeutic approaches to chronic hyperuricemia and gout. High Blood Press Cardiovasc Prev.

[48] Goicoechea M, Garcia de Vinuesa S, Verdalles U (2010). Effect of allopurinol in chronic kidney disease progression and cardiovascular risk. Clin J Am Soc Nephrol.

[49] Tsuruta Y, Nitta K, Akizawa T (2014). Association between allopurinol and mortality among Japanese hemodialysis patients: results from the DOPPS. Int Urol Nephrol.

[50] Yelken B, Caliskan Y, Gorgulu N (2012). Reduction of uric acid levels with allopurinol treatment improves endothelial function in patients with chronic kidney disease. Clin Nephrol.

[51] Beberashvili I, Erlich A, Azar A (2016). Longitudinal study of serum uric acid, nutritional status, and mortality in maintenance hemodialysis patients. Clin J Am Soc Nephrol.

