# Supplementary material for: Serum uric acid and cardiovascular mortality in chronic kidney disease: a meta-analysis
Source: BMC Nephrol. 2019 Jan 14;20:18. doi: 10.1186/s12882-018-1143-7 (PMC6330757; doi:10.1186/s12882-018-1143-7)
Supplement: Supplementary file 1 — Table S1. The reasons of excluded articles at the stage of eligibility. The list showed the reasons of excluded articles. (DOCX 86 kb) [file 12882_2018_1143_MOESM1_ESM.docx]

Additional file 1: Table S1 The reasons of excluded articles at the stage of eligibility.

| Author, year | Journal origin | The reasons of exclusion |
| --- | --- | --- |
| Hsu, 2004 | Nephrol Dial Transplant.2004;19(2):457-62. | The outcome was all-cause mortality. No outcome of interest. |
| Suliman, 2006 | Am J Kidney Dis.2006;48(5):761-71. | The outcome was all-cause mortality. No outcome of interest. |
| Chien, 2008 | Atherosclerosis.2008;197(2):860-867. | No outcomes in chronic kidney disease patients. |
| Ojie, 2008 | American Journal of Kidney Disease,2008;51:A76. | The outcome was all-cause mortality. No outcome of interest. |
| Weiner, 2008 | Am J Kidney Dis.2008;51(2):212-23. | The outcomes were cardiovascular events and all-cause mortality. No outcome of interest. |
| Lee, 2009 | Am J Nephrol.2009;29(2):79-85. | The outcome was all-cause mortality. No outcome of interest. |
| Navaneethan, 2009 | Nephrol Dial Transplant.2009(24):1260-1266 | The outcomes were cardiovascular events and all-cause mortality. No outcome of interest. |
| Ekundayo, 2010 | Int J Cardiol.2010;142(3):279-87. | The outcomes were cardiovascular events and all-cause mortality. No outcome of interest. |
| Kowalczyk, 2010 | Nephron Clin Pract. 2010;116(2):c114-22. | The outcome was all-cause mortality. No outcome of interest. |
| Filippatos, 2011 | Eur Heart J.2011;32(6):712-20. | No enough data to estimate hazard ratio. No response to the email for additional data. |
| Neri, 2011 | Am J Kidney Dis.2011;58(3):398-408. | The outcomes were cardiovascular events. No outcome of interest. |
| Ito, 2012 | Hypertens Res.2012;35(8):867-73. | The outcomes were cardiovascular events. No outcome of interest. |
| Autunovic, 2013 | Int Urol Nephrol.2013(45):1111-1119. | No enough data to estimate hazard ratio. Analysis the association between 1umol/l uric acid increase and cardiovascular mortality. |
| Chung, 2013 | Am J Nephrol.2013;37(5):452-61. | The outcome was all-cause mortality. No outcome of interest. |
| Dawson, 2013 | Hypertension.2013;62(1):105-11. | No outcomes in chronic kidney disease patients. |
| Feng, 2013 | Kidney Blood Press Res.2013;37(2-3):181-9. | The outcome was all-cause mortality. No outcome of interest. |
| Gouri, 2013 | Pak J Biol Sci.2013;16(17):852-8. | The article analyzed the relationship between serum uric acid and cardiovascular risks. No outcome of interest. |
| Wu, 2013 | BMC Nephrology.2013;22(14):92. | The outcome was all-cause mortality. No outcome of interest. |
| Jeon, 2014 | J Ren Nutr.2014;24(5):336-42. | The outcome was all-cause mortality. No outcome of interest. |
| Odden, 2014 | Am J Kidney Dis.2014;64(4):550-7. | No outcomes in chronic kidney disease patients. |
| Vaduganathan, 2014 | American Journal of Cardiology.2014;114(11):1713-1721. | No outcomes in chronic kidney disease patients. |
| Xia, 2014 | Am J Kidney Dis.2014;64(2):257-64. | Multiple reports of the same subpopulation (Xia, 2016). |
| Chen, 2015 | Ann Rheum Dis.2015;74(11):2034-42. | The outcome was all-cause mortality. No outcome of interest. |
| Lazzeri, 2015 | Scand Cardiovasc J.2015;49(1):14-9. | The outcome was all-cause mortality. No outcome of interest. |
| Park, 2015 | Hypertension Research.2015;38(6):433-438. | The expose was metabolic acid load. No exposure of interest. |
| Shimizu, 2015 | American Journal of Physiology-Heart and Circulatory Physiology.2015;309(7):H1123-H1129. | No outcomes in chronic kidney disease patients. |
| Beberashvili, 2016 | Clin J Am Soc Nephrol.2016;29.pii: CJN.10400915.[Epub ahead of print]. | Multiple reports of the same subpopulation (Beberashvili, 2015).  Measurement of serum uric acid concentration every 6 months. Time-varing serum uric acid not baseline serum uric acid was examined. |
| Nacak, 2015 | Nephrology Dialysis Transplantation.2015;30(12)2039-2045. | The outcome was decline in renal function. No outcome of interest. |
| Bae,2016 | Medicine(Baltimore).2016:95(24):e3701. | The outcome was all-cause mortality. No outcome of interest. |
| Huang, 2016 | Circulation Journal.2016;80(2):404-410. | No outcomes in chronic kidney disease patients. |
| Kanbay, 2016 | Int Urol Nephrol.2016 Mar 23.[Epub ahead of print]. | The outcomes were cardiovascular events and all-cause mortality. No outcome of interest. |
| Park, 2017 | Nephrol Dial Transplant. 2017 Jan 7. [Epub ahead of print]. | The outcome was all-cause mortality. No outcome of interest. |
| Longecker, 2017 | BMC Nephrol.2017;18 (1):103. | The outcome was all-cause mortality. No outcome of interest. |
