# Supplementary figures and images for: Serum uric acid and cardiovascular mortality in chronic kidney disease: a meta-analysis
Source: BMC Nephrol. 2019 Jan 14;20:18. doi: 10.1186/s12882-018-1143-7 (PMC6330757; doi:10.1186/s12882-018-1143-7)

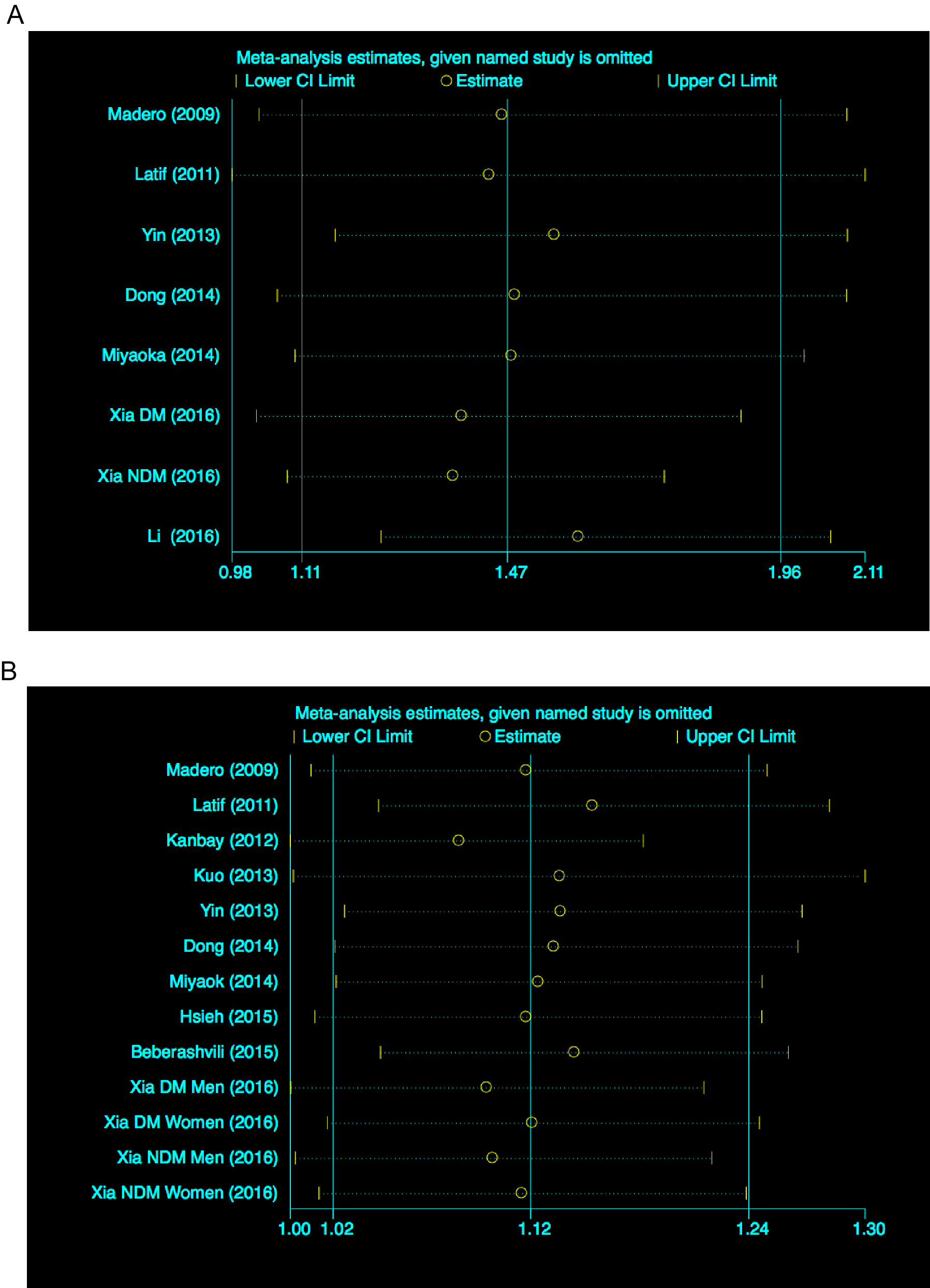

Supplement: Supplementary file 2 — Figure S1. Sensitivity analysis. Sensitivity analyses were performed to evaluate the influence of a signal study on the overall risk estimate by removing one study at a time. (DOCX 13200 kb) [file 12882_2018_1143_MOESM2_ESM.docx]

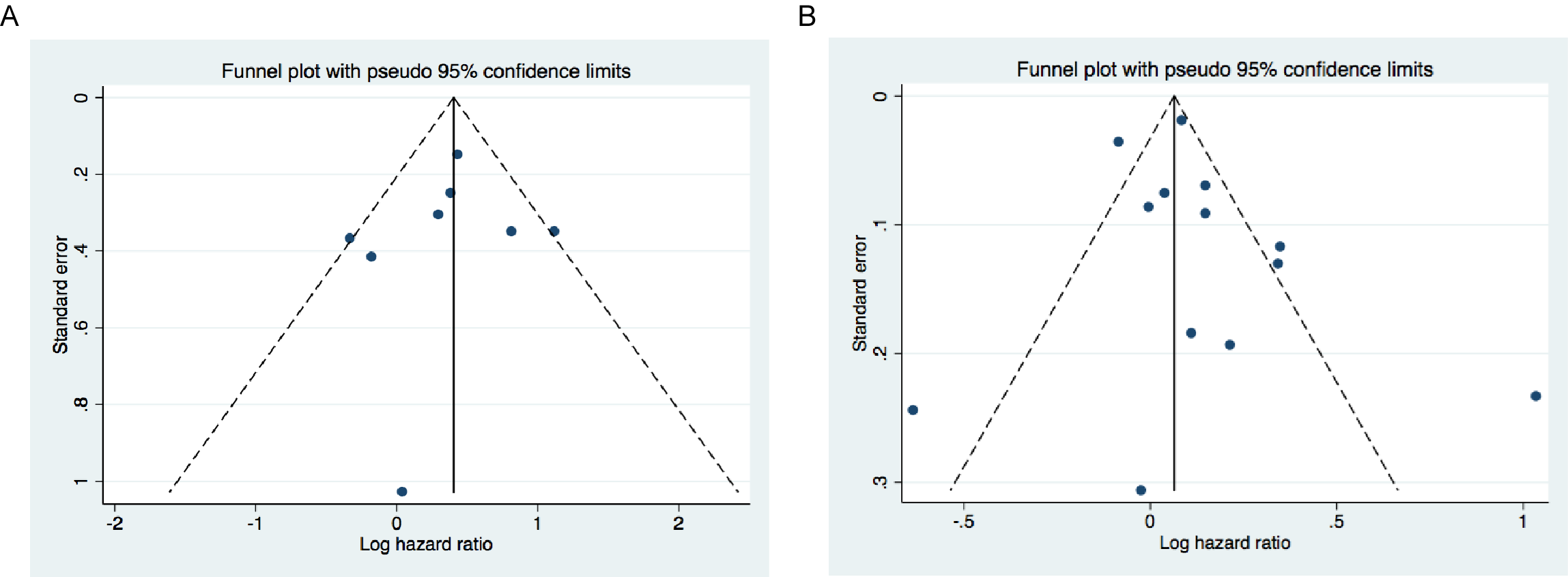

Supplement: Supplementary file 3 — Figure S2. Publication bias. Funnel plots and Egger’s test were performed to assess the potential publication bias. (DOCX 5726 kb) [file 12882_2018_1143_MOESM3_ESM.docx]
